# Novel non-invasive ECG imaging method based on the 12-lead ECG for reconstruction of ventricular activation: A proof-of-concept study

**DOI:** 10.3389/fcvm.2023.1087568

**Published:** 2023-02-02

**Authors:** Patricia Zerlang Fruelund, Peter M. Van Dam, Jacob Melgaard, Anders Sommer, Søren Lundbye-Christensen, Peter Søgaard, Tomas Zaremba, Claus Graff, Sam Riahi

**Affiliations:** ^1^Department of Cardiology, Aalborg University Hospital, Aalborg, Denmark; ^2^Department of Clinical Medicine, Aalborg University, Aalborg, Denmark; ^3^Department of Cardiology, University Medical Center Utrecht, Utrecht, Netherlands; ^4^Department of Health Science and Technology, Faculty of Medicine, Aalborg University, Aalborg, Denmark; ^5^Unit of Clinical Biostatistics, Aalborg University Hospital, Aalborg, Denmark

**Keywords:** electrocardiography, non-invasive imaging, cardiac pacing, ventricular activation, patient-specific modeling, 12-lead electrocardiogram

## Abstract

**Aim:**

Current non-invasive electrocardiographic imaging (ECGi) methods are often based on complex body surface potential mapping, limiting the clinical applicability. The aim of this pilot study was to evaluate the ability of a novel non-invasive ECGi method, based on the standard 12-lead ECG, to localize initial site of ventricular activation in right ventricular (RV) paced patients. Validation of the method was performed by comparing the ECGi reconstructed earliest site of activation against the true RV pacing site determined from cardiac computed tomography (CT).

**Methods:**

This was a retrospective study using data from 34 patients, previously implanted with a dual chamber pacemaker due to advanced atrioventricular block. True RV lead position was determined from analysis of a post-implant cardiac CT scan. The ECGi method was based on an inverse-ECG algorithm applying electrophysiological rules. The algorithm integrated information from an RV paced 12-lead ECG together with a CT-derived patient-specific heart-thorax geometric model to reconstruct a 3D electrical ventricular activation map.

**Results:**

The mean geodesic localization error (LE) between the ECGi reconstructed initial site of activation and the RV lead insertion site determined from CT was 13.9 ± 5.6 mm. The mean RV endocardial surface area was 146.0 ± 30.0 cm^2^ and the mean circular LE area was 7.0 ± 5.2 cm^2^ resulting in a relative LE of 5.0 ± 4.0%.

**Conclusion:**

We demonstrated a novel non-invasive ECGi method, based on the 12-lead ECG, that accurately localized the RV pacing site in relation to the ventricular anatomy.

## 1. Introduction

Along with QRS morphology from the 12-lead electrocardiogram (ECG), fluoroscopy is the main guiding tool for right ventricular (RV) pacemaker lead implantation. However, several studies have demonstrated the inaccuracy of both fluoroscopy and QRS morphology to determine RV lead implantation site (1–4). Interindividual variations in cardiac and thoracic anatomy, underlying myocardial pathology and variability in ECG electrode positioning influence the recorded ECG waveforms (5). Additionally, the activation patterns resulting from RV pacing at different lead positions may result only in subtle morphological QRS differences making interpretation difficult and thus reducing the usefulness of the 12-lead ECG to guide pacemaker implantation (3, 4). The inaccuracy of current implantation methods result in a high risk of unintended RV free wall implantations (2, 6). Therefore, new methods are needed to ensure optimal RV lead implantation.

Non-invasive electrocardiographic imaging (ECGi) provides 3D reconstruction of the cardiac electrical activity overcoming some of the limitations from the standard 12-lead ECG, potentially increasing the diagnostic value (7). However, ECGi methods require specialized technical equipment to obtain detailed mapping of body surface potentials using a dense array of electrodes placed on the patients thorax (8). Thus, the usefulness of current ECGi methods is limited and not easily implemented in everyday clinical practice (9, 10).

The aim of this pilot study was to evaluate the ability of a novel non-invasive ECGi method, based on the standard 12-lead ECG, to localize initial site of ventricular activation in RV paced patients. Validation of the method was performed by comparing the earliest site of activation estimated from the reconstructed ventricular activation model against the true RV pacing site determined from cardiac computed tomography (CT).

## 2. Materials and methods

### 2.1. Study population

Thirty-four patients who underwent *de novo* implantation of a dual chamber pacemaker due to advanced atrioventricular block at Aalborg University Hospital between December 2014 and December 2017 were retrospectively included. Patients with suspected fusion pacing or patients who had been upgraded to cardiac resynchronization therapy after primary pacemaker implantation were not considered eligible. Following data were acquired at time of study inclusion: clinical characteristics from electronic medical records, a contrast-enhanced cardiac CT scan, an RV paced 12-lead ECG including a 3D photography showing the ECG electrode positions and a transthoracic echocardiography.

### 2.2. Cardiac CT acquisition

If no contrast-enhanced cardiac CT, showing the RV lead position, was available (*n* = 30), a study specific cardiac CT was acquired using a second-generation dual source scanner (Siemens Somatom Definition Flash, Siemens Healthcarem Erlangen, Germany). During breath hold, the contrast-enhanced retrospective ECG gated scan was timed with contrast filling of both ventricular cavities. Reconstruction was done in diastole with a mean slice thickness of 0.95 ± 0.1 mm for the narrow field of view focused on the heart. The mean total dose length product (DLP) for the study-acquired CT scans was 114.9 ± 65.3 mGy cm and mean contrast dose (Iopromide 370 mg/ml) was 67.4 ± 9.6 ml.

A segmental approach was used to retrospectively categorize RV lead position (2). The RV long axis was divided into equal thirds and subsequently the RV lead position was analyzed in a short axis view. Lead position was categorized to be apical if located on the lower third of the RV septum. Lead position was categorized as septal if located on the upper two thirds of the RV septum. Leads positioned at the anteroseptal or posteroseptal junction were considered septal. Leads were categorized as free wall if positioned on the RV free wall regardless of long axis position.

### 2.3. Echocardiography

All patients had a transthoracic echocardiography performed at time of study inclusion using a 2.5-MHz transducer on a commercially available ultrasound system (VIVID E95, GE Healthcare, Milwaukee, WI, USA). The echocardiograms were analyzed using EchoPAC software (GE Healthcare, Milwaukee, WI, USA). Analyses included estimation of left ventricular ejection fraction (LVEF) and left ventricular (LV) volumes (11).

### 2.4. The 12-lead ECG based ECGi method

**FIGURE 1:**
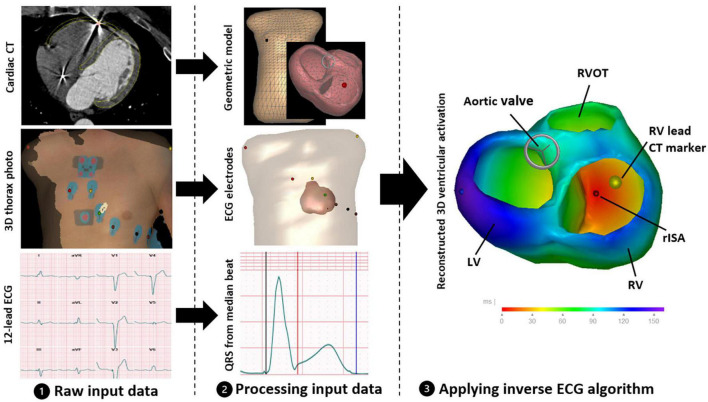
Summary of the stepwise process used for reconstructing 3D ventricular activation. (1) Collecting the raw input data including contrast-enhanced cardiac CT, 3D thorax photograph documenting the ECG electrode positions and 12-lead ECG with right ventricular pacing. (2) Processing the input data including creation of a patient-specific geometric model with ECG electrodes correctly positioned on the thorax model and defining QRS onset and duration using the 12-lead ECG median beat. (3) Integration of processed input data using the inverse ECG algorithm creating the 3D ventricular activation model. LV, left ventricle; RV, right ventricle; RVOT, right ventricular outflow tract; rISA, reconstructed initial site of activation (rISA).

The ECGi method was based on an inverse-ECG algorithm that reconstructs the electrical activity of the ventricles by integrating information from an RV-paced 12-lead ECG, location of surface ECG electrode positions together with a CT-derived patient-specific heart-thorax geometric model (12). The electrical activation was reconstructed in a stepwise approach including collection of input data, processing input data and finally applying the inverse ECG algorithm (Figure 1).

For each patient, a standard 12-lead ECG during RV pacing was digitally recorded at time of study inclusion using the Cardiovit AT-102 plus resting ECG machine (Schiller, Baar, Switzerland). All ECG recordings were imported to the MUSE Cardiology Information System (GE Healthcare, Wauwatosa, WI, USA). In MUSE, QRS onset and thus duration was manually adjusted on median beats formed by version 23 of the Marquette 12SL algorithm to exclude the pacing spike. A single QRS onset was defined across the 12 leads as the earliest positive or negative deflection after a pacing spike. Furthermore, a 3D photography of the patient thorax was recorded documenting the position of the ECG electrodes at time of the ECG recording.

The patient specific heart-thorax geometric models were created from the cardiac CT scan using specialized research software (GeomPeacs, Peacs BV, Netherlands) (13). The model creation process was semi-automated, morphing a standard model to match the contours of the patient-specific cardiac and thoracic anatomy. The geometry was constructed as a triangular surface mesh with discrete nodes contouring the four heart valves, the endocardial and epicardial borders of the left and right ventricles and the thoracic walls. The 3D thorax photography of the ECG electrodes was merged with the thorax model, to correctly position the ECG electrodes on the thorax model. Furthermore, also using the specialized DICOM software, the CT images were used to localize the RV lead implantation site and a marker was placed at the endocardial border of the heart geometry where the RV lead touched the RV endocardium.

**FIGURE 2:**
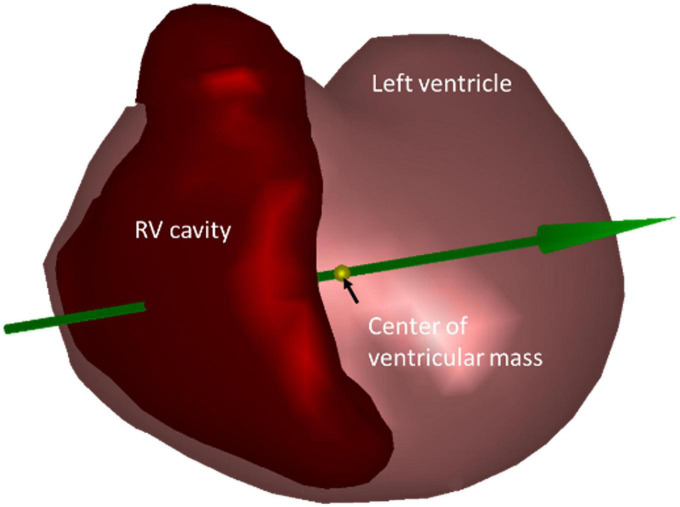
Geometric model of the heart showing the mean QRS axis (green arrow) located at the center of ventricular mass (yellow marker). The QRS axis crossing points are where the green arrow enters and exits the right ventricular (RV) cavity.

Application of the algorithm was automated using specialized software (Supplementary material). The algorithm workflow, in short, was as follows:

a) The first step was to determine the QRS axis. The heart-thorax model was used to derive the vectorcardiogram (VCG) from the recorded 12-lead ECG, using the method described by Boonstra et al. (14). The mean QRS-axis was localized to the center of ventricular mass. Entry and exit points were then defined as the QRS axis crossing points of the right ventricular cavity in the heart model (Figure 2).

b) The Fastest Route Algorithm, together with a realistic myocardial propagation velocity (0.7–0.85 m/s), was used to compute depolarization times between the discrete nodes on the closed triangulated modeled myocardial surface mesh (15). In this algorithm, the anisotropic nature of the myocardial tissue was captured by a 2.5 times slower transmural velocity than the velocity over the ventricular surface (16). The current problem at hand required the localization of the RV stimulation site, thus limiting the search space for initial site of activation to just the RV endocardium. In the current approach the initial search for the RV lead stimulation site was restricted to the two QRS axis RV crossing points. Using the Fastest Route Algorithm, QRS durations were simulated initiating from discrete nodes around the two QRS axis crossing points and the discrete node resulting in the simulated QRS duration that best matched the recorded QRS duration was selected to be the initial estimate for RV stimulation site.
c) The cardiac depolarization sequence was used to compute ECGs applying the equivalent dipole layer model as a source model, where local transmembrane potentials simulated the local currents generated by the heart (17, 18). The effect of the constructed volume conductor model was computed using the boundary element method previously described (19, 20). Assigned conductivity values were 0.2 S/m for the thorax and ventricular muscles, and 0.6 S/m for the blood cavities.
d) Subsequently, the improvement procedure was started in which additional extra late break through points (foci) were added in the left cavity, mimicking (late) breakthroughs from the His-Purkinje system. The location and timing of the initial RV stimulation site and the added His-Purkinje nodes was optimized in an iterative procedure, matching the recorded ECG and simulated ECGs. The simulated ECGs were the result of the depolarization sequence generated from the location and timing of the combination of different foci.
e) Finally, the depolarization sequence resulting in a simulated ECG best matching the recorded ECG was chosen for final analysis.

### 2.5. Validation

**FIGURE 3:**
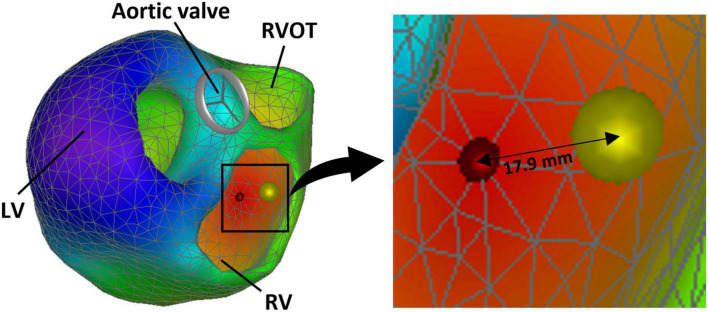
Reconstructed 3D ventricular electrical activation sequence showing the reconstructed initial site of activation (rISA) (red marker) and the right ventricular (RV) lead position assessed from computed tomography (CT) (yellow marker). The localization error (LE) is the geodesic distance between the center of the CT marker and the estimated initial site of activation. LV, left ventricle; RVOT, right ventricular outflow tract.

Performance of the reconstructed electrical activation was assessed by calculating the localization error (LE) defined by the geodesic distance (shortest distances between two points on a curved surface) between the RV CT-marker and the reconstructed initial site of activation (rISA) (Figure 3). Furthermore, a circular area calculated using the LE as the radius was compared to the RV endocardial surface area to demonstrate the LE relative to the RV endocardial size.

The rISA was constrained to the discrete nodes of the ventricular geometric model whereas the marker for RV lead position was localized on the CT scan and imported into the heart model without this constraint. This created a potential default distance between the RV marker and the rISA. This was adjusted for by calculating a corrected localization error (cLE) defined as the distance from the rISA to the discrete node closest to the RV marker on the path between the rISA and the RV marker. A cLE of 0 mm meant that rISA was located in the triangle to where the RV lead marker was projected on to the endocardial model surface.

Performance of the reconstructed activation sequence was assessed comparing it with an activation sequence forced to initiate from the known RV lead insertion site. This was done to assess how sensitive the overall activation sequence was to change in initial site of activation. Lastly, the correlation between the recorded ECG and the ECG fitting the reconstructed model was assessed.

### 2.6. Statistical analyses

Continuous variables are reported as mean ± standard deviation (range) unless severely skewed. Categorical values are reported as absolute numbers and percentages. For analyzing associations, unpaired *t*-test was used for binary independent variables and fractional polynomial regression was used for continuous independent variables. To compare different activation maps within one patient the Pearson correlation coefficient was used. *p* < 0.05 was considered statistically significant. STATA version 17 was used to perform the statistical analyses.

## 3. Results

### 3.1. Study cohort and characteristics

Patient characteristics (Table 1) showed that 20 (58.8%) were male with a mean age of 69.1 ± 11.9 years (range 24.9–84.7 years) at time of study inclusion and a mean duration of pacemaker treatment of 3.4 ± 0.8 years (range 2.6–5.5 years). All patients had a long RV paced QRS duration with a mean duration of 151.2 ± 12.9 ms (range 128–182 ms).

**TABLE 1:** Patient characteristics.

	All (*n* = 34)
Age (years)	69.1 ± 11.9 (24.9–85.0)
Male	20 (58.8)
Duration of pacemaker treatment (years)	3.4 ± 0.8 (2.6–5.5)
Ischemic heart disease	4 (11.8)
Beta-blockers	17 (50.0)
Body mass index (kg/m^2^)	29.5 ± 5.9 (19.6–48.2)
**Echocardiographic parameters**	
Left ventricular ejection fraction (%)	55.4 ± 7.6 (41.7–71.4)
Left ventricular end-diastolic volume (ml)	101.9 ± 28.3 (64.0–187.0)
**Right ventricular paced ECG parameters**	
QRS duration (ms)	151.2 ± 12.9 (128.0–182.0)
Heart rate (beats per minute)	66.5 ± 8.5 (50.0–86.0)

Values are expressed as mean ± standard deviation (range) or *n* (%).

### 3.2. Localization error of reconstructed initial site of activation

**FIGURE 4:**
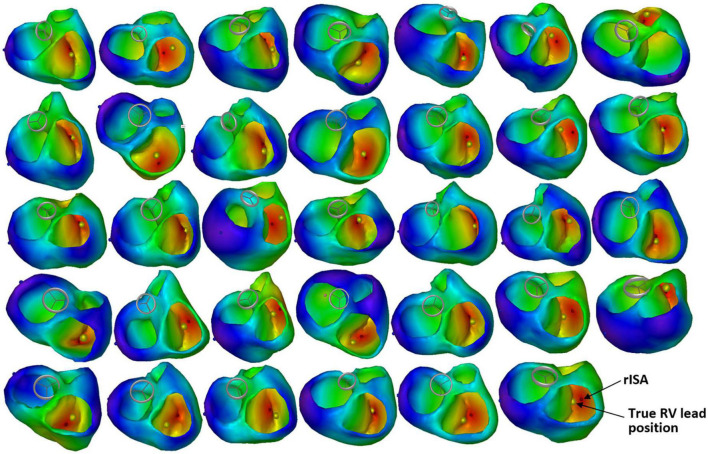
Reconstructed 3D ventricular activation models for all 34 patients. The red marker indicates the reconstructed initial site of activation (rISA), and the yellow marker indicates the true right ventricular (RV) lead position determined by computed tomography (CT).

**FIGURE 5:**
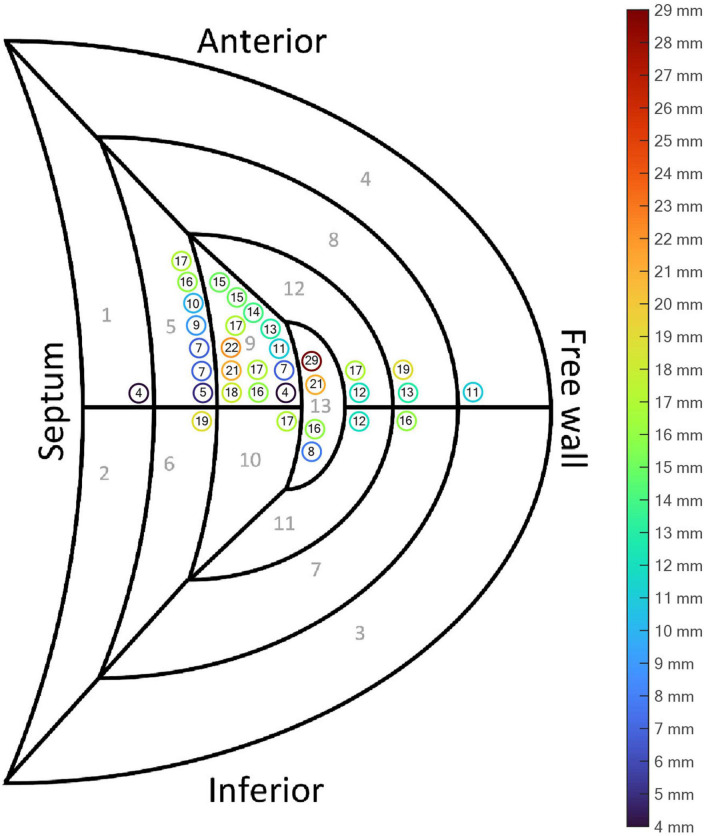
Segmental plot of the localization error (LE) according to the right ventricular (RV) lead position. The encircled numbers show the LE (mm) for each patient. The color of the circle is a visual representation of the distance.

The mean LE was 13.9 ± 5.6 mm (range 4.3–28.6 mm) (Table 2 and Figures 4, 5). For 17 (50.0%) of the 34 patients, the LE was < 15.0 mm and for 30 (88.2%) of the patients the LE was < 20.0 mm. There were four patients with an LE above 20.0 mm including one outlier with an LE of 28.6 mm. The mean distance from the RV CT marker to the nearest discrete node on the path to rISA was 4.3 ± 2.2 mm (range 1.0–8.7 mm). Correcting for this, the mean cLE was 9.6 ± 6.2 mm (range 0.0–24.6 mm). The mean RV endocardial surface area was 146.0 ± 30.0 cm^2^ and the mean circular LE area was 7.0 ± 5.2 cm^2^ resulting in a LE error of 5.0 ± 4.0% relative to the RV endocardial area.

**TABLE 2:** Results.

Patient	RV lead location	QRS duration (ms)	LE (mm)	cLE (mm)	RV area (cm^2^)	Relative LE (%)	Correlation (*r*)
1	Septal	156	8.7	0.0	178.3	1.3	0.98
2	Free wall	156	17.3	15.2	154.3	6.1	0.98
3	Apical	158	20.6	15.1	165.0	8.1	0.87
4	Apical	142	15.6	13.7	167.9	4.6	0.88
5	Apical	158	6.6	4.3	158.0	0.9	0.84
6	Free wall	170	13.4	11.7	173.2	3.2	0.92
7	Free wall	148	11.3	10.3	129.2	3.1	0.96
8	Septal	134	7.1	0.0	146.6	1.1	0.97
9	Free wall	160	12.0	8.2	122.6	3.7	0.94
10	Septal	132	9.5	6.6	125.6	6.0	0.97
11	Free wall	182	7.2	0.0	179.8	2.7	0.98
12	Apical	140	10.4	6.9	126.7	2.9	0.90
13	Septal	168	16.5	10.4	147.0	5.8	0.88
14	Septal	148	4.5	0.0	135.1	0.5	0.97
15	Apical	152	4.4	0.0	169.1	0.4	0.91
16	Apical	142	21.2	15.8	123.1	11.5	0.80
17	Free wall	172	17.9	10.9	158.6	7.1	0.73
18	Septal	130	7.2	0.0	168.2	1.0	0.92
19	Apical	164	18.0	14.3	246.0	4.1	0.94
20	Apical	156	14.6	8.3	172.4	3.9	0.98
21	Apical	156	12.4	5.4	173.7	4.9	0.96
22	Apical	128	7.5	4.5	104.3	1.9	0.98
23	Apical	166	15.2	13.7	118.6	6.1	0.90
24	Apical	138	16.7	12.9	97.5	9.0	0.99
25	Free wall	142	16.3	14.5	159.5	5.2	0.94
26	Apical	152	19.1	16.5	155.1	5.7	0.91
27	Apical	152	21.5	15.5	149.6	9.7	0.89
28	Septal	146	4.3	0.0	96.0	0.6	0.96
29	Septal	146	10.1	6.2	111.6	2.9	0.94
30	Septal	150	18.7	17.5	139.5	7.9	0.80
31	Apical	156	28.6	24.6	124.8	20.6	0.84
32	Apical	146	12.7	4.5	149.4	3.4	0.93
33	Apical	162	19.6	18.2	123.0	7.7	0.91
34	Apical	132	14.5	9.4	114.8	5.7	0.95
Mean ± SD		151.2 ± 12.9	13.9 ± 5.6	9.6 ± 6.2	146.0 ± 30.0	5.0 ± 4.0	0.92 ± 0.1

LE, localization error; cLE, corrected localization error; RV, right ventricular; SD, standard deviation.

Several variables possibly affecting local cardiac conduction properties, myocardial propagation velocity and quality of the input data were investigated for their potential to affect the accuracy of the localization algorithm. Following variables were tested for an association with LE: body mass index, gender, age, use of beta-blocker, ischemic heart disease, LVEF, LV end-diastolic volume, RV lead segmental position, noise in the recorded ECG, heart rate and QRS duration. Only the segmental RV lead position was significantly associated with the LE. The mean uncorrected LE among those with a non-septal lead position was 15.2 mm (95% CI 13.1–17.4) compared to 10.3 mm (95% CI 6.2–14.4, *p* = 0.02) among those with a septal lead position. This association remained significant for the cLE and in a sensitivity-analysis excluding the outlier.

### 3.3. Assessment of the activation sequence

**FIGURE 6:**
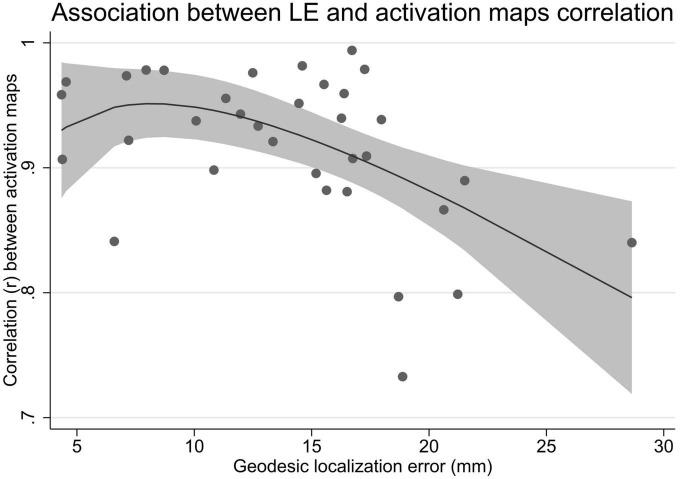
Association between the localization error and the correlation between initially reconstructed activation sequence and the activation sequence initiating from the true right ventricular lead position. LE, localization error.

The correlation between the initial reconstructed activation sequence and the activation sequence initiating from the RV CT marker position was 0.92 ± 0.06 (range 0.73–0.99) (Table 2). The correlation between activation maps was associated with the uncorrected and cLE, with increasing LE resulting in decreasing correlation between activation maps, *p* = 0.01Check if the edit made in line 1062 is fine.. The correlation dropped significantly for LE > 18 mm (Figure 6). Mean correlation for LE < 18 mm was 0.94 ± 0.04 and for LE > 18 mm mean correlation was 0.82 ± 0.06.

### 3.4. Accuracy of the fitted ECG

Overall, the fitted ECG was highly correlated with the recorded ECG [median *r* = 0.88 (range 0.62–0.99)]. Correlation was > 0.8 in 28 (82%) patients. The correlation between the fitted ECG and the recorded ECG was not associated with the LE (*p* = 0.22).

### 3.5. Computation time

Having loaded the patient-specific model and 12-lead ECG into the specialized software system, the mean algorithm computation time used for reconstructing the ventricular activation was 1.1 ± 0.4 s per ECG using a standard laptop (Intel CORE i7 CPU).

## 4. Discussion

In this pilot study, we developed and validated a novel non-invasive ECGi method based on the 12-lead ECG for reconstructing ventricular activation during RV pacing and showed: (1) The rISA was accurately and effectively localized in relation to ventricular anatomy. (2) The reconstructed ventricular activation sequence was robust to small changes in initial site of activation.

Current ECGi methods are often based on more complex body surface potential mapping (BSM) derived from up to 256 leads (10). Only few studies have investigated 12-lead ECG based methods similar to the method used in this study (21). This is, to our knowledge, the first 12-lead ECG based method tested using clinical data from RV paced patients. Previous studies have validated BSM-based ECGi-methods by assessing the LE between known pacing sites and rISA. Oosterhoff et al. found an average LE of 18 mm using pig hearts and 64-lead BSM (22). Using the smaller rabbit hearts, Han et al. found an LE of approximately 5 mm using 64-lead BSM (23). A study from 2015 used data from 29 pacemaker patients and found a mean LE of 8.6 mm using 224-lead BSM (24). In our study, using only the 10 standard ECG electrodes, we found a LE of 13.9 mm, which is comparable to the studies using multiple lead BSM.

It was not possible to evaluate the accuracy of the ECGi method using invasive electrocardiographic mapping as ground truth data, as it was not available for comparison (9). However, the RV lead position determined from CT provided a reliable fix point for comparison. The robustness of the activation sequence was tested comparing the initial reconstructed activation sequence and the activation sequence initiated from the RV CT marker position. As would be expected, we found an overall strong correlation (*r* = 0.92, range 0.73–0.99) indicating that despite varying degrees of LE, the reconstructed activation pattern is robust to smaller changes in initial site of activation. The used regularization method in the final optimization, minimizing the L2 error norm of the ECG signals, maximizes the correlation between measured and simulated ECG signals. Due to the smoothness of the initial estimate based on a physiological based activation pattern, no large variations are expected in the error function. Consequently, the found initial correlations are equivalent to the resulting fitting errors in the modeled activation pattern. However, it was not possible to compare the reconstructed activation sequence with invasive electrophysiological mapping and the accuracy of the reconstructed activation sequence needs to be confirmed in future studies using invasive electrocardiographic mapping for comparison.

ECGi is not a direct measurement of electrical activity of the heart, it is a reconstruction estimated by an algorithm using different input data. Regardless of the ECGi method used, there is some inherent sources of error depending on the algorithm used and the quality of the input data (9, 25, 26). In this study, ECG-gated CT scans were reconstructed in diastole. Cardiac motion and changes in size and myocardial thickness during the cardiac cycle results in small inaccuracies in the heart-thorax model when assessing ventricular activation during systole. Furthermore, several patients had incomplete thoracic borders on the CT scan, making the thorax model and positioning of the ECG electrodes less accurate. However, we investigated a wide range of variables with potential to affect the accuracy of the localization algorithm and only RV lead position seemed to significantly affect the LE. The mean LE for non-septal leads was higher than for the septal leads (Table 2 and Figure 5). Leads located in the RV free wall or apex are closer to the body surface, and thus become more sensitive to the exact position of the ECG electrodes and local inhomogeneities like for instance the ribcage and lungs. The latter structures were not part of the reconstructed used volume conductor model. The spatial distribution of the 12-lead ECG may be too coarse to localize the exact position of RV leads on the free wall. Moreover, the volume conductor model is inaccurate, both in assigned general homogeneous conductivity values as well as the fact that the heart moves during contraction and the breathing cycle, which potentially influences the RV free wall more than the septum which is located in the middle of the heart. However, though the LE was larger for the non-septal RV leads, the LE was still small, and the method in general seems robust despite imperfections in the algorithm and the input data. Importantly, the method seems to be sensitive enough to distinguish between RV free wall and septal position (Figure 4).

Our ECGi method assumed a uniform conduction velocity throughout the myocardium. However, we do not know how well the method works when the assumptions in the algorithm are violated. Elderly pacemaker patients often suffer from ischemia, diabetes or hypertension with hypertrophic myocardium and fibrosis resulting in areas of slower conduction velocities (27, 28). This is accounted for indirectly, as the estimated QRS duration must be matched with the recorded QRS duration. However, detailed assessment of the activation sequence localizing ischemic or fibrotic areas with slow conduction is not possible with the current method. Despite this, the method showed to accurately localize the initial site of activation in a heterogenous cohort including both males and females with a wide range of QRS durations, body sizes, ages, LV functions and sizes as well as RV leads positioned throughout the RV endocardium.

Based on these promising results, further work to improve the accuracy of the method seems encouraging. The reconstructions are calculated almost real-time. Thus, potentially enabling intraprocedural model-assisted evaluation of ventricular activation and lead position in relation to ventricular anatomy ensuring optimal pacemaker lead implantation for the individual patient. Furthermore, providing a detailed electrical activation pattern, ECGi is highly relevant as a tool to guide and optimize cardiac resynchronyzation therapy (CRT) implantation (9, 29–31).

### 4.1. Limitations

As described in detail above, comparison with ground truth data from invasive electrocardiographic mapping was not available and the quality of the input data was varying possibly limiting the accuracy. The current method includes creating a patient-specific heart-thorax model, which is a cumbersome process requiring a cardiac CT scan. Future studies should investigate the feasibility of using a generic model to reduce the workload and avoid the need for a CT scan, making the method more manageable in a clinical setting.

## 5. Conclusion

In this pilot study, we demonstrated a novel non-invasive ECGi method, based on the readily available 12-lead ECG, which accurately and effectively localized initial site of activation in relation to ventricular anatomy and provided an estimate for the ventricular activation sequence during RV pacing.

## Data availability statement

The raw data supporting the conclusions of this article will be made available by the authors, without undue reservation.

## Ethics statement

Ethical review and approval was not required for the study on human participants in accordance with the local legislation and institutional requirements. The patients/participants provided their written informed consent to participate in this study.

## Author contributions

PF: conceptualization, investigation, methodology, formal analysis, data curation, writing—original draft, and visualization. PV: conceptualization, methodology, software, visualization, and writing—review and editing. AS, JM, and TZ: conceptualization and writing—review and editing. SL-C: conceptualization, formal analysis, and writing—review and editing. PS: investigation and writing—review and editing. CG: conceptualization, methodology, data curation, and writing—review and editing. SR: conceptualization, methodology, writing—review and editing, and supervision. All authors contributed to the article and approved the submitted version.
