# Supplementary material for: Novel non-invasive ECG imaging method based on the 12-lead ECG for reconstruction of ventricular activation: A proof-of-concept study
Source: Front Cardiovasc Med. 2023 Feb 2;10:1087568. doi: 10.3389/fcvm.2023.1087568 (PMC9932809; doi:10.3389/fcvm.2023.1087568)
Supplement: Supplementary file 1 [file Image_1.pdf]

Background

Solving the inverse problem requires a forward model to compute the potentials on the body surface using a 3D description of Ohms law: U = I x R, where R is the volume conductor model, where the 3D resistivity is estimated by the boundary element method, and I follows the local transmembrane potential. In this study, we are only investigating the depolarization of the heart, i.e. the upstroke of the transmembrane potential, assuming the transmembrane is constant after depolarization. Therefore, there is a direct relation between depolarization moment and generation of current (1). The effect of the constructed volume conductor model was computed using the boundary element method previously described (2). Assigned conductivity values were 0.2 S/m for the thorax and ventricular muscles, and 0.6 S/m for the blood cavities.

The algorithm workflow is shown in Figure A. The subsequent sections describe each step in detail.

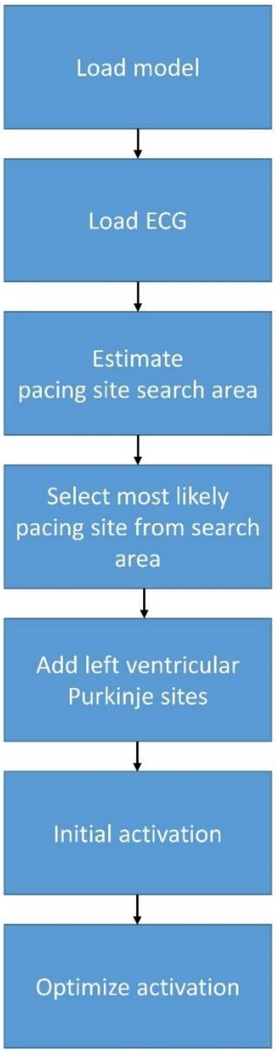

Figure A. Algorithm workflow outlining each step of the process.

Load model

The model contains:

- 3D triangulated mesh of the heart, blood cavities, and torso
- A boundary element matrix that converts local transmembrane potentials to potentials on the body surface (2)

- 3D distance matrices for distances within the heart including a distance matrix in which the transmural distances are virtually made longer to correct for the slower trans-fibre propagation velocity (3,4)
- The location of the ECG electrodes on the body surface. When an ECG electrode is located within a triangle the potential is linearly interpolated within the triangle based on the computed ECG signals on the vertices of the triangles.

Load ECG

Load the measured ECG, the one that needs to be matched by the simulated ECG signals

Estimate pacing site search area

Compute the vectorcardiogram in a simplified version to van Dam et al. and Boonstra et al. (5,6):

In detail: The $\overrightarrow{VCG}$, the direction of activation, is computed from the 9 electrodes, building the 12-lead ECG by the following equation:

$$\overrightarrow{VCG}(t) = \sum_{el=1}^{9} ecg_{el}(t) \cdot \alpha_{el} |\, r_{el} \,| \qquad eq. 1$$

where $|\, r_{el} \,|$ is the normalized vector between the 3D-position of the mass centre of the heart and the electrode position on the thorax (r_{el}). The $ecg_{el}(t)$ is the value of the ECG at an electrode at time-sample t. Factor α_{el} was set to 0 for the x direction and 2 for the *y* and *z* directions for the unaugmented extremity leads (VR, VL, and VF), for all other leads α_{el} was set to 1.

The mean QRS-axis was also localized to the centre of ventricular mass. Entry and exit points were then defined as the QRS axis crossing points of the right ventricular cavity in the heart model (Figure B).

Based on this QRS axis a search zone is determined by the entry and exit point of the QRS axis. Nodes of the ventricular triangulated mesh within 25 mm from this line will be part of the search zone.

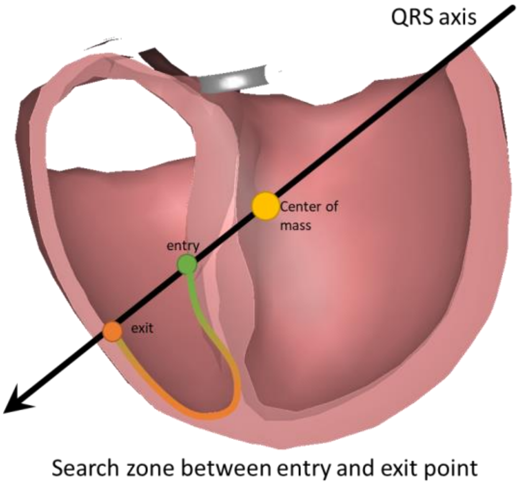

Figure B Localization of the search zone based on the QRS axis. The zone indicated by the green – orange line between entry and exit point of the QRS axis in the right chamber determines the search zone.

Select most likely pacing site from search area

Every node within the search area is used to compute an activation sequence by means of the fastest route algorithm. A realistic myocardial propagation velocity (0.7-0.85 m/s) was used to compute depolarization times between the discrete nodes on the closed triangulated modelled myocardial surface mesh (3). In this algorithm, the anisotropic nature of the myocardial tissue was captured by a 2.5 times slower transmural velocity than the velocity over the ventricular surface (7). The site with a near matching QRS duration and best matching simulated ECG with the measured ECG is selected as the initial estimate for the pacing site.

Add left ventricular Purkinje sites

In some cases, the His-Purkinje system can be activated retrogradely from right stimulation site to the left side (Figure C). As this system is faster than the myocardial propagation three extra foci are added on the left side. The timing is set to a representative timing given the distance from the stimulation site and the assumed timed the His-Purkinje system can be picked up.

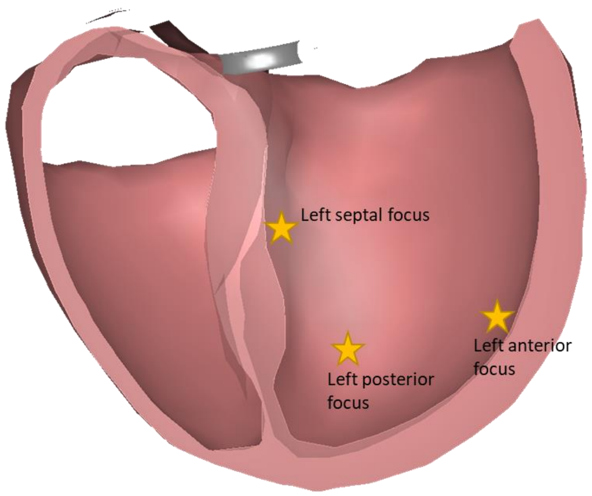

Figure C. The added left sided foci associated with the left side of the His-Purkinje system; left septal, anterior, and posterior focus.

Initial activation

Adjust position and timing of the four foci, one in the right ventricle and three in the left ventricle, to obtain the best matching ECG. The combination of four individual activation sequences are determined by using the first activation that reaches any of the ventricular nodes. For this purpose, the timing and position is shifted slightly in an iterative procedure. For the position the fastest route algorithm distance from any neighbouring node is tested and the resulting ECGs are compared to the measured ECG. The node with the highest Pearson correlation is selected as the new focus position. Similar for the timing, the timing is made 1 ms early or later. The ECG that produces a higher correlation to the measured ECG is selected as the newly shifted timing.

Optimized Activation

Finally, the depolarization sequence resulting in a simulated ECG best matching the recorded ECG was chosen for final analysis. For this purpose, a dedicated Levenberg-Marquardt optimization scheme is used with the Laplacian of the local activation times as a regularization operator (7,8).

References

1. Boonstra MJ, Oostendorp TF, Roudijk RW, Kloosterman M, Asselbergs FW, Loh P, Van Dam PM. Incorporating structural abnormalities in equivalent dipole layer based ECG simulations. *Front Physiol* (2022) 13:1089343. doi: 10.3389/fphys.2022.1089343

2. Meijs JW, Weier OW, Peters MJ, van Oosterom A, Oosterom AVAN. On the Numerical Accuracy of the Boundary Element Method. *IEEE Trans Biomed Eng* (1989) 36:1038–1049. doi: 10.1109/10.40805

3. van Dam PM, Oostendorp TF, van Oosterom A. Application of the fastest route algorithm in the interactive simulation of the effect of local ischemia on the ECG. *Med Biol Eng Comput* (2009) 47:11–20. doi: 10.1007/s11517-008-0391-2

4. Dam PM van, Oostendorp TF, Oosterom A van. Interactive simulation of the activation sequence: Replacing effect by cause. *2011 Computing in Cardiology*. (2011). p. 657–660

5. Van Dam PM, Locati ET, Ciconte G, Borrelli V, Heilbron F, Santinelli V, Vicedomini G, Monasky MM, Micaglio E, Giannelli L, et al. Novel CineECG Derived from Standard 12-Lead ECG Enables Right Ventricle Outflow Tract Localization of Electrical Substrate in Patients with Brugada Syndrome. *Circ Arrhythmia Electrophysiol* (2020)1007–1018. doi: 10.1161/CIRCEP.120.008524

6. Boonstra MJ, Brooks DH, Loh P, van Dam PM. CineECG: A novel method to image the average activation sequence in the heart from the 12-lead ECG. *Comput Biol Med* (2022) 141:105128. doi: 10.1016/j.compbiomed.2021.105128

7. Van Dam PM, Oostendorp TF, Linnenbank AC, Van Oosterom A. Non-invasive imaging of cardiac activation and recovery. *Ann Biomed Eng* (2009) 37:1739–1756. doi: 10.1007/s10439-009-9747-5

8. Marquardt DW. An Algorithm for Least-Squares Estimation of Nonlinear Parameters. *J Soc Ind Appl Math* (1963) 11:431–441. http://www.jstor.org/stable/2098941
